# Centrality and interhemispheric coordination are related to different clinical/behavioral factors in attention deficit/hyperactivity disorder: a resting-state fMRI study

**DOI:** 10.1007/s11682-022-00708-8

**Published:** 2022-07-21

**Authors:** Livio Tarchi, Stefano Damiani, Teresa Fantoni, Tiziana Pisano, Giovanni Castellini, Pierluigi Politi, Valdo Ricca

**Affiliations:** 1 Psychiatry Unit, Department of Health Sciences, University of Florence, Florence, FI Italy; 2 Department of Brain and Behavioral Science, University of Pavia, 27100 Pavia, Italy; 3 Pediatric Neurology, Neurogenetics and Neurobiology Unit and Laboratories, Neuroscience Department, Meyer Children’s Hospital, University of Florence, Florence, Italy

**Keywords:** Attention Deficit/Hyperactivity Disorder (ADHD), Resting-state fMRI, Eigenvector Centrality (EC), Voxel-Mirrored Homotopic Connectivity (VMHC), Brain development

## Abstract

Eigenvector-Centrality (EC) has shown promising results in the field of Psychiatry, with early results also pertaining to ADHD. Parallel efforts have focused on the description of aberrant interhemispheric coordination in ADHD, as measured by Voxel-Mirrored-Homotopic-Connectivity (VMHC), with early evidence of altered Resting-State fMRI. A sample was collected from the ADHD200-NYU initiative: 86 neurotypicals and 89 participants with ADHD between 7 and 18 years old were included after quality control for motion. After preprocessing, voxel-wise EC and VMHC values between diagnostic groups were compared, and network-level values from 15 functional networks extracted. Age, ADHD severity (Connor’s Parent Rating-Scale), IQ (Wechsler-Abbreviated-Scale), and right-hand dominance were correlated with EC/VMHC values in the whole sample and within groups, both at the voxel-wise and network-level. Motion was controlled by censoring time-points with Framewise-Displacement > 0.5 mm, as well as controlling for group differences in mean Framewise-Displacement values. EC was significantly higher in ADHD compared to neurotypicals in the left inferior Frontal lobe, Lingual gyri, Peri-Calcarine cortex, superior and middle Occipital lobes, right inferior Occipital lobe, right middle Temporal gyrus, Fusiform gyri, bilateral Cuneus, right Precuneus, and Cerebellum (FDR-corrected-*p* = 0.05). No differences were observed between groups in voxel-wise VMHC. EC was positively correlated with ADHD severity scores at the network level (at *p*-value < 0.01, Inattentive: Cerebellum rho = 0.273; Hyper/Impulsive: High-Visual Network rho = 0.242, Cerebellum rho = 0.273; Global Index Severity: High-Visual Network rho = 0.241, Cerebellum rho = 0.293). No differences were observed between groups for motion (*p* = 0.443). While EC was more related to ADHD psychopathology, VMHC was consistently and negatively correlated with age across all networks.

**Supplementary Information:**

The online version contains supplementary material available at 10.1007/s11682-022-00708-8.

## Introduction

Attention Deficit/Hyperactivity disorder (ADHD) is characterized by symptoms presenting in a heterogeneous manner across individuals, including attention deficits, impulsivity, and hyper-activity (American Psychiatric Association, 2013). Functional Magnetic Resonance Imaging (fMRI) proved to be a powerful tool for exploring the neurobiological correlates of ADHD symptoms and behaviors (Damiani et al., 2020; Iravani et al., 2021; Qian et al., 2018; Rosch et al., 2018; Silva et al., 2021; Tarchi et al., 2021). Specifically, fMRI highlighted the importance of how each region is functionally connected to the rest of the brain. Two independent measures of these connections are centrality and interhemispheric coordination, the present study aims to elucidate their role in ADHD.

### Centrality measures

An important proxy used in fMRI analyses is centrality, a group of graph-theory based parameters which measure the degree of connection between a specific brain region and all others. fMRI and centrality measurements have allowed researchers to identify functional centers in moderating ADHD presentations and symptomatology (Damiani et al., 2020; Iravani et al., 2021; Rosch et al., 2018). The concept of functional centers considers brain regions as “nodes”, and each relationship between pairs of regions as “edges”. As centrality measurements quantify the number and strength of relationships between edges and nodes, functional centers are nodes with a high number of meaningful connections, that is, a high number of connections above a certain threshold. This conceptualization provides an efficient and simple instrument to better explore the complex functional organization of the brain, also known as the functional connectome (Iturria-Medina et al., 2008; Sporns, 2006; Sporns et al., 2005, 2007). Centrality measurements proved to have the ability to capture intrinsic features of the human functional connectome in both neurotypicals (Achard et al., 2006; He et al., 2009; Sporns et al., 2007; Tarchi et al., 2021; Zuo et al., 2012), and individuals with neuropsychiatric disorders (Reinelt et al., 2019; Seidel et al., 2020), including ADHD (M. Zhou et al., 2019). However, the available evidence showed both increased and decreased centrality scores in ADHD compared to neurotypical controls, in particular for the superior Temporal lobes and the middle/inferior Occipital lobes (Di Martino et al., 2013; Hong et al., 2017; Tarchi et al., 2021; Zhou et al., 2019). Analyses of the age contribution to centrality measurements in ADHD indicated a role for development in moderating the Resting-State fMRI activity in the middle Temporal cortex (Hong et al., 2017), with additional reports of transient alterations during development among patients with ADHD (Damiani et al., 2020; Hong et al., 2017). However, recent literature in the field of Computational Psychiatry and fMRI has focused the attention on subcortical structures (Castellanos et al., 2008; Damiani et al., 2020; Giraldo-Chica & Woodward, 2017; Lottman et al., 2019; Zhou et al., 2017), and preliminary evidence highlighted their key role in ADHD (Bruchhage et al., 2018; Damiani et al., 2020). For these reasons, a centrality measurement sensitive to the contribution of subcortical structures was preferred in the current study. When compared to other centrality measurements (e.g. Degree of Centrality), Eigenvector Centrality (EC) proved to be more sensitive subcortical regions (Zuo et al., 2012), and was thus selected as the centrality measurement of choice, also considering its recursive nature (Lohmann et al., 2010).

### Interhemispheric coordination

Parallel efforts in the study of the intrinsic characteristics of the human brain, as assessed by fMRI, have focused on the degree of functional integration between hemispheres, i.e. their interhemispheric coordination (Halpern et al., 2005). Interhemispheric coordination has been defined as the degree of left–right symmetry in the brain activity. Lower interhemispheric coordination has concerned a number of functions and associated brain areas, at the molecular, cellular, and functional level (with relevance of asymmetry both during Resting-State, Toga & Thompson, 2003; and task conditions, Riès et al., 2016). The clinical relevance of increased or decreased hemispheric specialization in individuals has not yet been fully elucidated, while multiple theories rely on atypical lateralization as a mechanism for the onset of neuropsychiatric disorders (Angrilli et al., 2009; Berretz et al., 2020; Vingerhoets, 2019).

For these reasons, Voxel-Mirrored Homotopic Connectivity (VMHC) was developed in order to assess the degree of homotopy in fMRI (that is, the degree of similarity between symmetric brain regions, Wei et al., 2018). VMHC has been shown to yield valuable insight on psychiatric conditions in Resting-State fMRI scans. In particular, a lower interhemispheric coordination has been reported in depression (Guo et al., 2013; L. Wang et al., 2013; Zhang et al., 2020); obsessive–compulsive disorder (Deng et al., 2019), schizophrenia (D. Wang et al., 2019), and bipolar disorder (L. Zhao et al., 2017). Although more commonly reported at the voxel-wise, whole-brain level, the characterization of brain networks by degree of interhemispheric coordination as assessed by VMHC has been proposed as a reliable marker of neurodegenerative processes (Cheung et al., 2021). The use of VMHC also seems supported by evidence of high test–retest stability (intraclass correlation coefficient ≥ 0.8, Dai et al., 2020), in contrast to other similar measurements of interhemispheric coordination (Hagemann et al., 2002).

For what concerns ADHD, current reports highlighted specific VMHC alterations in this population (Jiang et al., 2014, 2019; Zhou et al., 2018). In particular, lower VMHC was found in children with ADHD in comparison to neurotypicals in the Occipital lobes. VMHC also negatively correlated with anxiety scores at the Conners’ Parent Rating Scale and positively correlated with set-shifting abilities in children with ADHD (J. Zhou et al., 2018). Contrasting evidence was offered by Jiang et al. (2019), who showed higher VMHC scores in children with ADHD in comparison to neurotypicals in the Occipital cortex. Our group recently suggested that age can partially explain these contrasting findings in ADHD fMRI, since cortical-subcortical connectivity can show transient alterations that are observable in specific time points between childhood and adulthood (Damiani et al., 2020).

### The current study

Neuroplasticity is known to shape brain development during late childhood, adolescence and early adulthood (Aoki et al., 2017; Guyer et al., 2018; Kadis et al., 2011; Petanjek et al., 2011; Selemon, 2013), and age-related changes have been observed in interhemispheric coordination or brain centrality during the same period of life in both clinical conditions and the general population (Anderson et al., 2011; Di Martino et al., 2013; Everts et al., 2009; Kadis et al., 2011; Lo et al., 2011; Nagel et al., 2013; Oades, 1998; Sato et al., 2015; Schneider et al., 2011; M. Zhou et al., 2019). A divergence of neurodevelopment has been postulated for ADHD (American Psychiatric Association, 2013), as, among other factors, individuals with ADHD report delays in language or social development more frequently than their peers (American Psychiatric Association, 2013; Bruce et al., 2006; Staikova et al., 2013). Therefore, a description of the patterns of neurodevelopment in individuals with ADHD and neurotypical controls is warranted for the interval between 7 and 18 years of age, a salient time span characterized by the onset of both ADHD (American Psychiatric Association, 2013; Chandra et al., 2021; Kieling et al., 2010; Rohde et al., 2000) and a relevant portion of all psychiatric disorders (Kessler et al., 2007a, b; Solmi et al., 2021).

### Aims

These premises call for using multiple whole brain, voxel-wise parameters which could explore brain connectivity in ADHD. Centrality and interhemispheric coordination may thus provide two different perspectives on ADHD brain connectivity: the former is more related to the global weight of a voxel, the second to the degree of symmetry reached between two homotopic voxels.

The primary aim of this study was to evaluate the potential differences in centrality (EC) and interhemispheric coordination of the brain (VMHC) in participants with ADHD, compared to neurotypicals, using a sample of adolescents between the age of 7 and 18 years old at the voxel-wise level.

Although previous studies focused on voxel-wise differences between ADHD and neurotypicals, the current work also adopted a network-based approach to provide novel insights on EC/VMHC. This approach allows to clearly visualize the relationship between neuroimaging and clinical findings (Tarchi et al., 2021), and to improve their replicability (Nickerson, 2018). The secondary aims of this study were i) to evaluate potential differences between neurotypicals and patients with ADHD in EC and VMHC at the network level. ii) to characterize the correlation of EC and VMHC with age, symptom severity, and cognitive/behavioral scores (Intelligence Quotient—verbal, performance, and full score; handedness—right hand dominance).

## Methods

### Sample

The current study sample was obtained from the New York University dataset of the ADHD200 repository, specifically from the International Neuroimaging Data-Sharing Initiative. All participants were between 7 and 18 years of age. A quality check for each subject was present in the phenotypic key provided with the dataset, and those subjects that did not pass were discarded preventively. The psychiatric diagnosis was based on the Schedule of Affective Disorders and Schizophrenia for Children—Present and Lifetime Version (Kaufman et al., 1997), administered to parents and children. ADHD specific psychopathology was evaluated through the Conners’ Parent Rating Scale-Revised, Long version (Gurley, 2011). Intelligence was evaluated with the Wechsler Abbreviated Scale of Intelligence (Canivez et al., 2009). Inclusion in the ADHD group required a diagnosis of ADHD based on parent and child responses to the Schedule of Affective Disorders and Schizophrenia for Children: Present and Lifetime Version, as well as on a T-score greater than or equal to 65 on at least one ADHD related index of the Conners’ Parent Rating Scale-Revised, Long version. Psychostimulant drugs were withheld at least 24 h before scanning. Inclusion criteria for the control group of neurotypicals required absence of any Axis-I psychiatric diagnoses per parent and child as per the interview by the Schedule of Affective Disorders and Schizophrenia for Children: Present and Lifetime Version, as well as T-scores below 60 for all the Conners’ Parent Rating Scale- Revised, Long version ADHD summary scales. Estimates of a Full Intelligence Quotient above 80, right-handedness and absence of other chronic medical conditions were required for all children (*ADHD200, *n.d.—NYU sample). Handedness was reported in a dimensional manner, and all included participants were right-handed. A handedness score ranging from 0 to + 1 was used to quantify the degree of right-hand dominance. Further details about the sample can be found in the parent study (Castellanos et al., 2008). MRI data was acquired in a single site (New York University, Child Study Center), and on one of two 3 T Siemens Trio scanners. Functional MRI scans were collected using a T2*-weighted echo-planar imaging (EPI) sequence with the following parameters: slice thickness 4 mm, repetition time 2 s, echo time 20 ms, flip angle 90°, voxel size 3.0 × 3.0x4.0 mm. An anatomical scan was acquired for each participant and defaced in order to preserve anonymity, the voxel size was 1.3 × 1.0x1.3 mm. The Resting-State fMRI scan lasted 304 s. Participants were asked to remain still and keep their eyes closed; they were not presented with stimuli or asked to respond during the scan.

### Preprocessing

fMRI data preprocessing steps were implemented in AFNI (Cox, 1996; Cox & Hyde, 1997; Taylor & Saad, 2013). Firstly, the structural and functional reference images were co-registered (Saad et al., 2013). The first 4 frames of each fMRI run were removed in order to discard the transient effects in amplitude observed until magnetization achieves steady state (Caballero-Gaudes & Reynolds, 2017). Slice timing correction (Konstantareas & Hewitt, 2001) and despike methods (Satterthwaite et al., 2013) were applied. Rigid-body alignment of the structural and functional image was performed. The anatomical image was then warped using the Montreal Neurological Institute (MNI) standard space template provided with the AFNI binaries. A symmetrical template was chosen in order to better compare results of inter-hemispheric connectivity. The “2009c” symmetric template of the MNI152 initiative was chosen as the template of choice. Volume registration was then used to align the functional data to the base volume, warping it to the stereotactic space of choice. Bandpass (0.01–0.1 Hz) was performed (Shirer et al., 2015). Each of the voxel time series was then scaled to have a mean of 100. To control for non-neural noise, regression based on the 6 rigid body motion parameters and their 6 derivatives was applied, as well as mean time series from cerebro-spinal fluid masks (Fox et al., 2005; Vovk et al., 2011) eroded by one voxel (Chai et al., 2012). Regression of white matter artifacts was performed through the fast ANATICOR technique as included in AFNI (Jo et al., 2010). To further improve motion correction, censoring of voxels with a Framewise Displacement (FD) above 0.5 mm was applied to the timeseries (Power et al., 2014).

A visual quality assessment of each scan was performed at the end of preprocessing. Alignment between the anatomical and Resting-State scan, alignment between Resting-State scan and the reference volume, motion control (censored timepoints < 10% and absolute movement in each of the 6 motion parameters < 2 mm translation and < 2° rotation) were inspected, and subjects excluded if at least one was altered.

### Primary aims, voxel-wise analysis

EC measures the importance of a node based on its connections to other important nodes (Bonacich, 1972, 2007). In fMRI, EC is based on both the number and the strength of connections between areas of the brain, with the most commonly used computational methods relying on correlation coefficients between voxels (Wink et al., 2012). Importance is assigned to voxels based on two factors: the raw number of meaningful connections (above a certain correlation coefficient threshold), and the degree of connection to highly connected hubs. Whole brain, voxel-wise EC values of Resting-State scans were measured using FASTCAT functionalities implemented in AFNI (Taylor & Saad, 2013). EC was measured by first calculating Pearson’s correlation coefficients for each pair of voxels in the brain. As no sparsity or threshold correction coefficient is currently established in the literature, Fast Eigenvector Centrality was used as the method of choice to determine the correlation matrix (Wink et al., 2012). Subsequently, eigenvectors were calculated determining the largest eigenvalue in the correlation matrix according to the formula:

$$Rv = \lambda v$$

where

R: represents the correlation matrix,
v: represents the eigenvector of the matrix, and the scalar λ its corresponding eigenvalue.

VMHC, on the other hand, is a measure of interhemispheric coordination between corresponding areas in fMRI (Wei et al., 2018). In other words, VMHC measures the level of symmetry, or correlation, between left/right pairs of voxels or brain areas. VMHC values were computed by calculating the Pearson’s correlation coefficients between each voxel and its interhemispheric counterpart in the mirrored symmetrical brain space. Thereafter, the correlation values were *z transformed* to improve normality: whole brain, voxel-wise VMHC maps were computed for each participant, then normalized using Fisher z-transformation (Zuo et al., 2010). The adopted formula for computing Z-transformed VMHC values was the following:

$$\frac{1}{2}ln \frac{(1+v)}{(1-v)}$$

where

v: represented voxel-wise VMHC values.

### Secondary aims, network-level analysis

To calculate age-related variations, each participant’s voxel-wise connectivity results was averaged within 15 networks. Masks for networks were obtained from the Functional Imaging in Neuropsychiatric Disorder Lab website – University of Stanford (Greicius & Eger, n.d.; Shirer et al., 2012). As the cerebellum plays an important role in ADHD (Bruchhage et al., 2018; Curtin et al., 2018; Ding & Pang, 2021; Miquel et al., 2019; Zhao et al., 2021), but was not included in the set of functional networks, a cerebellar map was retrieved from previous studies on cerebellar segmentation in the MNI stereotactic space (Diedrichsen et al., 2009). In total, 15 networks were included in secondary analyses. These 15 masks include: Anterior Salience, Auditory, Basal Ganglia, dorsal Default Mode Network (DMN), high Visual, Language, Left Executive Control, posterior Salience, Precuneus, Primary Visual, Right Executive Control, Sensorimotor, ventral DMN, and Visuospatial networks, as well as a Cerebellar mask. A graphical representation of network maps is offered by the original publication from which the functional networks were derived (Shirer et al., 2012). In order to compare means between neurotypicals and patients with a diagnosis of ADHD, Student’s t-tests were calculated for mean EC/VMCH value per network, Hedges’ g estimate of effect size reported. Correlation coefficients were estimated between the mean EC/VMHC value in each network and age/symptoms scores. Correlation coefficients were also estimated between the mean EC/VMHC value per network and IQ scores or handedness.

### Control analyses

To control for the role of motion, group differences in mean FD values per run were explored through a student t-test, the estimated effect size was reported by Hedges’ g. A violin plot was used to graphically inspect group distributions in mean FD values, with a jitter element to represent individual observations. Quartile values per group were rendered in the distribution curve (25, 50, 75 percentiles).

### Statistical analyses

For both EC and VMHC, t-tests were used to measure whole brain, voxel-wise differences between neurotypicals and patients with a diagnosis of ADHD (3dttest +  + , by AFNI, Cox, 1996), with a False Discovery Rate corrected threshold (FDR-corrected-p) of 0.05. Significant voxels after thresholding were reported after clustering in order to remove potential, isolated, artifacts. A minimum cluster of 30 voxels with 3 Nearest Neighbors (NN) was selected in accordance with previous literature (Damiani et al., 2020). Age, sex, IQ (verbal, performance, full scores) and handedness were introduced as covariates when estimating group differences, using the 3dttest + AFNI command and the “-covariates” option. Results were also clustered according to standard practice, with minimum size of 30 voxels, calculated by the 3 nearest neighbors. Secondary analyses were conducted with R, version 4.1.2 (R Core Team, 2020) and its library *tidyverse* (Wickham et al., 2019). Correlation coefficients were estimated using Spearman’s rho, p-values reported via correlation matrices. Analyses on the full sample were repeated considering neurotypicals and ADHD groups separately. To account for multiple comparisons, a p-value of 0.01 was adopted as a significance threshold, while thresholds between 0.01 and 0.05 were referred to as trends in reporting the results.

## Results

### Descriptive Statistics

In the sample, 37 participants were excluded for excessive motions or quality control (9 TYP, 28 ADHD). 10 participants were excluded as at least one network had an EC value of 0, as it was not possible to calculate the respective value for computational or technical impossibility (4 TYP, 6 ADHD). In fact, current EC estimation methods are memory intensive and might not resolve the matrix operations (Taylor & Saad, 2013; Wink et al., 2012). A final count of 86 neurotypicals and 89 participants with ADHD were included in the study. Sample descriptives for both groups and overall can be found in Table 1.

**Table 1:** Descriptive statistics

	Neurotypicals	ADHD	Difference	Overall
N	86	89	/	175
Age	12.23 (± 3.10)	11.22 (± 2.76)	W 4522 * p 0.026	11.71 (± 2.97)
Handedness	0.62 (± 0.24)	0.66 (± 0.26)	W 3350 p 0.236	0.64 (± 0.25)
Gender	40 ♂ 46 ♀	66 ♂ 23 ♀	W 2651 * *p* < 0.001	106 ♂ 69 ♀
ADHD Global Index Severity	45.50 (± 6.34)	71.20 (± 8.53)	W 103 * *p* < 0.001	58.66 (± 14.92)
Inattentive score	45.55 (± 6.14)	70.44 (± 8.81)	W 122 * *p* < 0.001	58.29 (± 14.61)
Hyper/Impulsive score	46.40 (± 5.42)	67.43 (± 12.20)	W 386 * *p* < 0.001	57.16 (± 14.18)
Full IQ	110.57 (± 14.38)	107.41 (± 14.26)	W 3840 p 0.149	108.92 (± 14.36)
Verbal IQ	110.98 (± 13.56)	108.06 (± 14.79)	W 3789 p 0.202	109.46 (± 14.38)
Performance IQ	107.74 (± 14.89)	104.70 (± 13.82)	W 3833 p 0.155	106.15 (± 14.38)

Differences evaluated by Mann–Whitney U-test as normality was not assumed. Values reported ± 1 Standard Deviation

W = Wilcoxon-Mann–Whitney two-sample rank-sum

### Primary results

**Fig. 1:**
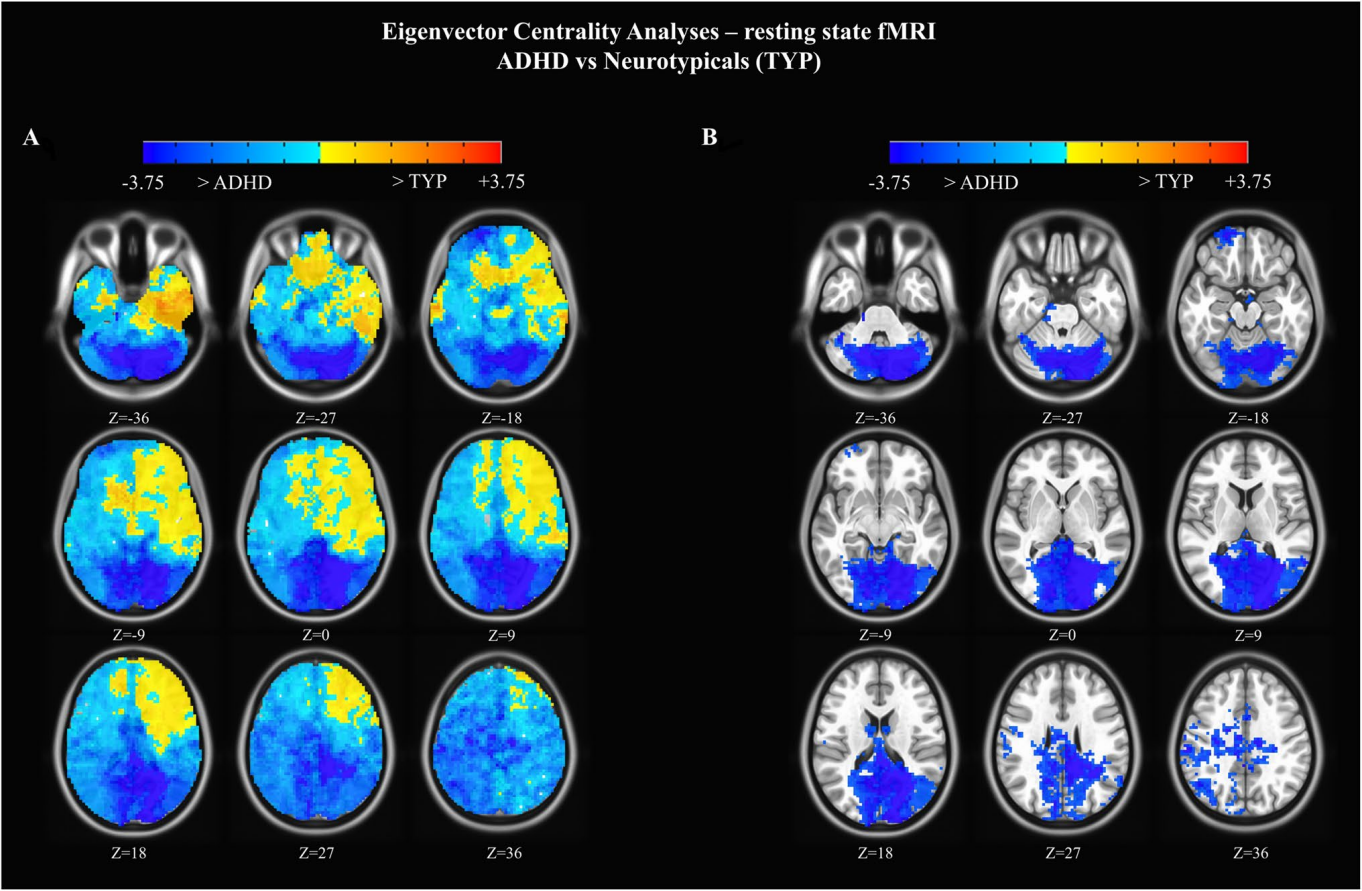
Voxel-wise results of Eigenvector Centrality analyses, no thresholding. Color-bar by Z-scores, from -3.75 to + 3.75, Blue higher in ADHD, Red higher in TYP. A. no thresholding, B. FDR-corrected-p 0.05 and minimum cluster size 30 voxels (NN 3)

Analysis of EC resulted in wide and diffuse differences between neurotypicals and ADHD participants, with results observed at a minimal FDR-corrected-p of 0.0005. A visual representation of non-thresholded results was reported in Fig. 1a, while a threshold of FDR-corrected-p 0.05 and a minimum of 30 voxel clusters (NN = 3) was used to represent results as Fig. 1b.

Significantly higher EC in ADHD compared to neurotypicals was found in the left inferior Frontal lobe, Lingual gyri, Peri-Calcarine cortex, superior and middle Occipital lobes, right inferior Occipital lobe, right middle Temporal gyrus, Fusiform gyri, bilateral Cuneus, right Precuneus, and Cerebellum. A detailed account of the thresholded clusters can be found in the Supplementary Materials as Table S1.

For what concerns individual factors, age, sex, clinical severity, and IQ scores did not appear to significantly influence between groups voxel-wise differences. Between groups, no covariate-map had surviving voxels at FDR-corrected-p 0.05.

Analysis of VMHC at the voxel-wise level resulted in no significant difference between neurotypicals and participants with ADHD, with no surviving voxel at FDR-corrected-p 0.05. Again, age, sex, clinical severity, and IQ scores did not appear to significantly influence between groups voxel-wise differences (no surviving voxels at FDR-corrected-p 0.05).

### Network-based analyses

Network-based analyses showed a significant difference in EC between neurotypicals (TYP) and ADHD patients in the Higher Visual, Primary Visual, Language and Posterior Salience Networks, as well as in the Cerebellum (higher EC among individuals with ADHD in all significant networks). VMHC did not show any significant difference in network-based analyses. Mean FD, as a measure of motion, was not significantly different between groups. Results were reported in Table 2.

**Table 2:** Network-based analyses, group differences between neurotypicals and ADHD

Network	t-statistic	*p*-value	Hedges' g
anterior_Salience_EC	1.162	0.247	0.178
Auditory_EC	0.115	0.909	0.020
Basal_Ganglia_EC	0.182	0.856	0.031
dorsal_DMN_EC	-1.266	0.207	-0.192
**high_Visual_EC**	**-3.704**	** < 0.001**	**-0.616**
**Language_EC**	**-2.082**	**0.039**	**-0.315**
LECN_EC	-0.228	0.820	-0.035
**post_Salience_EC**	**-3.113**	**0.002**	**-0.470**
Precuneus_EC	-1.055	0.293	-0.162
**prim_Visual_EC**	**-3.160**	**0.002**	**-0.539**
RECN_EC	-1.277	0.203	-0.196
Sensorimotor_EC	-1.309	0.192	-0.198
ventral_DMN_EC	-1.458	0.147	-0.223
Visuospatial_EC	-1.294	0.197	-0.195
**Cerebellum_EC**	**-4.229**	** < 0.001**	**-0.692**
anterior_Salience_VMHC	-1.065	0.288	-0.161
Auditory_VMHC	-0.468	0.641	-0.071
Basal_Ganglia_VMHC	-0.886	0.377	-0.134
dorsal_DMN_VMHC	-1.164	0.246	-0.176
high_Visual_VMHC	0.246	0.806	0.037
Language_VMHC	-1.130	0.260	-0.171
LECN_VMHC	0.638	0.524	0.096
post_Salience_VMHC	-0.231	0.817	-0.035
Precuneus_VMHC	-0.218	0.827	-0.033
prim_Visual_VMHC	-0.268	0.789	-0.041
RECN_VMHC	-0.346	0.730	-0.052
Sensorimotor_VMHC	-1.142	0.255	-0.172
ventral_DMN_VMHC	-0.405	0.686	-0.061
Visuospatial_VMHC	0.680	0.498	0.103
Cerebellum_VMHC	-1.192	0.235	-0.180
Mean FD	-0.769	0.443	-0.116

In bold, statistically significant results

*LECN* Left executive control network

*RECN* Right executive control network

**Fig. 2:**
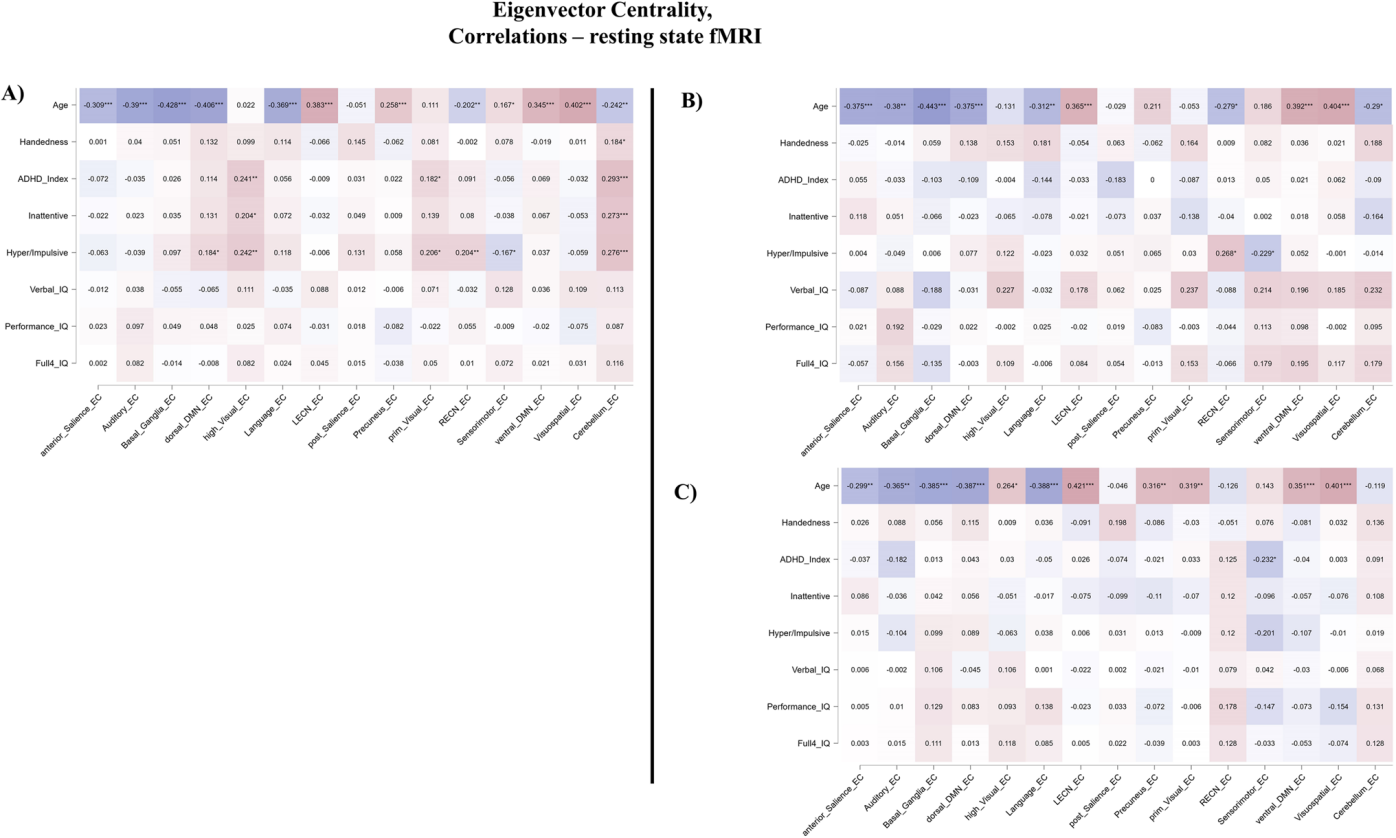
Heatmaps of Eigenvector Centrality correlations with age, handedness, IQ scores and severity scales. Colors from blue to red. DMN: Default Mode Network, LECN: Left Executive Control Network, RECN: Right Executive Control Network, Blue higher negative correlation coefficient, Red higher positive correlation coefficients. A: Heatmap of the overall sample, B: Heatmap for neurotypicals, TYP, C: Heatmap for patients with a diagnosis of ADHD, * *p*-value < 0.05, ** *p*-value < 0.01, *** *p*-value < 0.001

Network-based analyses showed a significant correlation between EC and age in 11 networks out of 15, when including all participants. Out of 15 networks, 7 showed a negative correlation between EC and age (Anterior Salience rho = -0.309; Auditory rho = -0.390; Basal Ganglia rho = -0.428; dorsal DMN rho = -0.406; Language rho = -0.369; Right Executive Control rho = -0.202; Cerebellum rho = -0.242), while 4 had a positive correlation (Left Executive Control rho = 0.383; Precuneus rho = 0.258; ventral DMN rho = 0.345; Visuospatial rho = 0.402). One network showed a positive trend between EC and age, namely the Sensorimotor network (rho = 0.167). Results for network-based analyses, including correlation coefficients and level of significance, were illustrated as Fig. 2. To be noted, EC values in the High Visual Network were positively correlated with age only for the ADHD group (ADHD rho = 0.264, *p*-value < 0.01; TYP rho = -0.131, *p*-value > 0.05; Overall rho = 0.022, *p*-value > 0.05).

Participants with ADHD showed a significant and negative trend between ADHD Global Index Severity and EC values in the Sensorimotor Network (rho = -0.232). For the overall sample, EC values also followed a positive trend with the Inattentive score of ADHD in the High Visual network (rho = 0.204) and a positive correlation with the Cerebellum (rho = 0.273). Additionally, a positive correlation was observed in the overall sample for the Hyper/Impulsive score in the High Visual (rho = 0.242), Right Executive Networks (rho = 0.204) and Cerebellum (rho = 0.276). Conversely, a negative trend was observed in the overall sample between EC values in the Sensorimotor Network and Hyper/Impulsivity severity scores (rho = -0.167). No significant correlation was found for EC and handedness or IQ, either as full or sub-domain scores. As previously reported, results for network-based analyses of EC correlation were illustrated as Fig. 2.

**Fig. 3:**
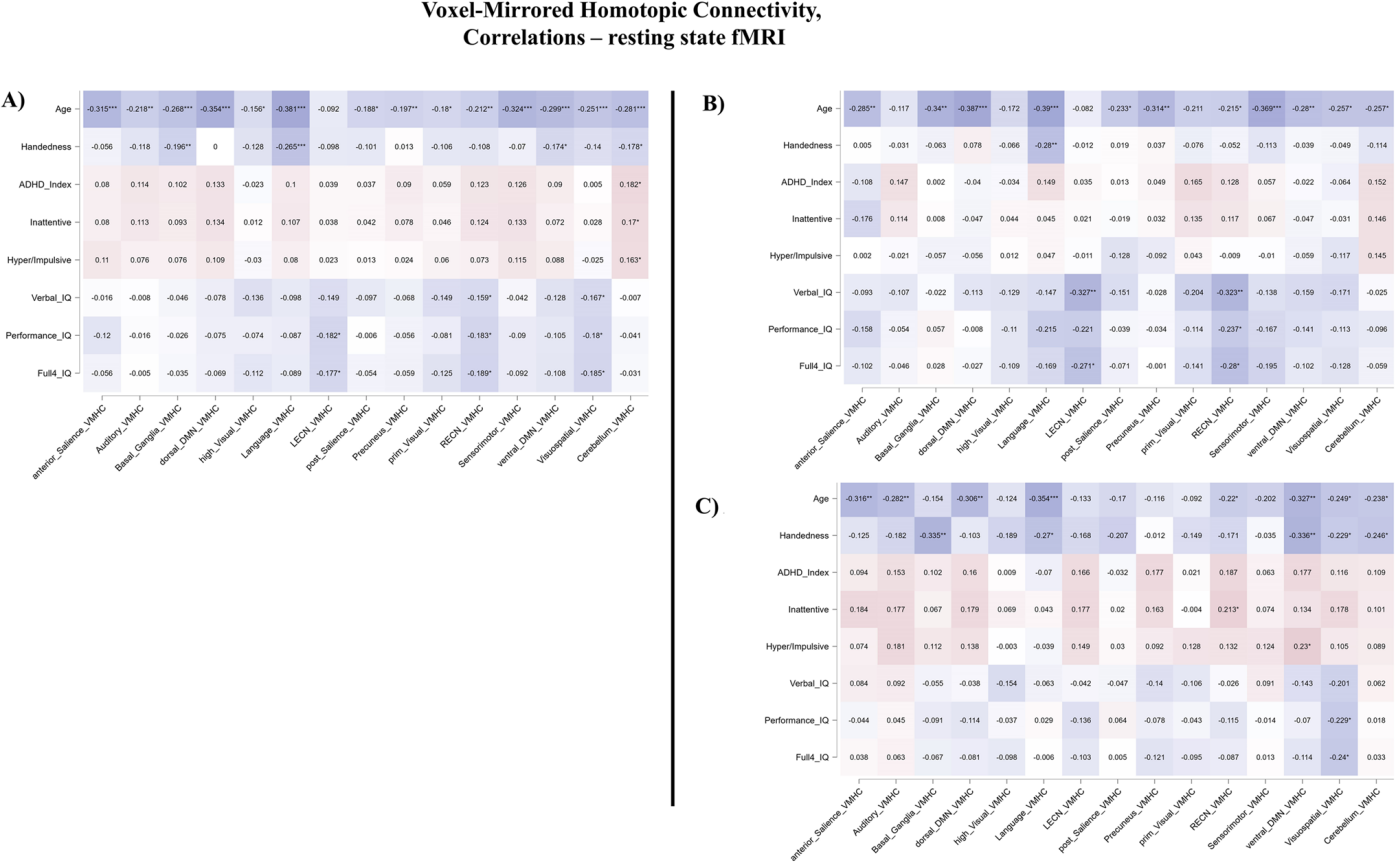
Heatmap of Voxel-wise Homotopic Connectivity correlations with age, handedness, IQ scores and severity scales. Colors from blue to red. DMN: Default Mode Network, LECN: Left Executive Control Network, RECN: Right Executive Control Network, Blue higher negative correlation coefficient, Red higher positive correlation coefficients. A: Heatmap of the overall sample, B: Heatmap for neurotypicals, TYP, C: Heatmap for patients with a diagnosis of ADHD, * *p*-value < 0.05, ** *p*-value < 0.01, *** *p*-value < 0.001

Network-based analyses showed a significant, negative correlation between VMHC and age in 11 out of 15 networks, when including all participants (Anterior Salience rho = -0.315; Auditory rho = -0.218; Basal Ganglia rho = -0.268; dorsal DMN rho = -0.354; Language rho = -0.381; Precuneus rho = -0.197; Right Executive Control rho = -0.212; Sensorimotor rho = -0.324; ventral DMN rho = -0.299; Visuospatial rho = -0.251; Cerebellum rho = -0.281). Results of the network-based analyses, including correlation coefficients and level of significance, were illustrated as Fig. 3.

Verbal IQ was negatively correlated with VMHC values in the Left and Right Executive Networks in neurotypicals (rho = -0.327 and rho = -0.323 respectively) but not among participants with ADHD. Performance IQ showed a negative trend only for the Right Executive Network in neurotypicals (rho = -0.237) and only with the Visuospatial Network in the group of patients with ADHD (rho = -0.229). Full IQ scores showed a negative trend with VMHC only in the Left and Right Executive Networks for neurotypicals (rho = -0.271 and rho = -0.280 respectively), while in the Visuospatial Network in the group of patients with ADHD (rho = -0.240). Handedness was negatively correlated with VMHC only in the Language Network for neurotypicals (rho = -0.280), while in Basal Ganglia and ventral DMN and for participants with ADHD (rho = -0.335 and rho = -0.336 respectively). Results for network-based analyses of VMHC correlations were illustrated in Fig. 3.

### Control analysis

No significant differences were observed for motion (mean FD value) between neurotypicals and participants with ADHD (*p* = 0.443, see Table 2). Violin plot distribution of mean FD value, with reported quartiles per group, showed high similarity and was illustrated in the Supplementary Materials as Supplementary Figure S2.

## Discussion

The present study confirms the importance of centrality measurements in the evaluation of psychiatric disorders. The observed increases of EC in ADHD in comparison to neurotypicals were in a wide area in the posterior half of the Cerebrum, including: the left inferior Frontal lobe, Lingual gyri, Peri-Calcarine cortex, superior and middle Occipital lobes, right inferior Occipital lobe, right middle Temporal gyrus, Fusiform gyri, bilateral Cuneus, right Precuneus, and Cerebellum. Although the current literature has focused on an aberrant interhemispheric coordination in ADHD, the current study did not find statistically significant differences between participants with ADHD and neurotypicals, as assessed by VMHC in a sample of participants aged from 7 to 18 years.

EC was particularly correlated with age at the network-level, pointing to a significant effect of neurodevelopment in the longitudinal trajectory of EC. Therefore, the present study offers a possible interpretation of the contrasting findings offered by previous literature. In fact, reports of increased centrality scores (Jiang et al., 2014) and decreased centrality scores (J. Zhou et al., 2018) in ADHD could be the result of specific alterations at different neurodevelopmental timepoints (Damiani et al., 2020; Hong et al., 2017). While early reports described increased centrality scores in ADHD for the superior Occipital lobes (M. Zhou et al., 2019), the current study observed a similar trend only for the inferior and medial Occipital lobes (Hong et al., 2017). Previous reports of increased centrality scores in ADHD for the Striatum, Pallidum, and Basal Ganglia (Di Martino et al., 2013) were not replicated. Furthermore, the current study supported decreased centrality scores in ADHD for the middle Temporal gyrus (Hong et al., 2017; Zhou et al., 2019).

Although age showed a homogeneous effect on VMHC (negative correlations in the overall sample and in each diagnostic group, across all networks), a heterogeneous correlation between EC and age was observed in the network-based analyses. Networks differentiated into three association patterns (positive, negative, or null), which remained similar when comparing analysis of single groups and across the entire sample. These trends can be interpreted in light of recent literature, which described different patterns of association between age and the structural/functional topography of the brain (Bellantuono et al., 2021; Long et al., 2017; Lopez-Larson et al., 2011; Zuo et al., 2012). For what concerns VMHC, only global patterns of interhemispheric coordination and development have been reported (Zuo et al., 2010). These patterns described a non-linear trend of decreasing global interhemispheric coordination before adulthood, and a later progressive increase after senility (Zuo et al., 2010). In previous literature, the right hemisphere exhibited higher values of EC as a function of age in comparison to its left homologue, as evaluated in a sample of healthy children aged 2 to 6 years old (Long et al., 2017). Associative areas, such as the right superior Frontal lobe and both superior/medial Temporal lobes, were observed to significantly increase in centrality scores as a function of age in the same sample (Long et al., 2017). Conversely, sensory areas such as the Occipital lobes and bilateral inferior Temporal lobes showed significantly decreased centrality scores with increasing age (Long et al., 2017). In the current study, the same areas (Occipital lobes—bilateral superior and middle gyri, right inferior gyrus; right middle Temporal gyrus) showed significantly higher EC in participants with ADHD aged between 7 to 18. In other words, the same areas which undergo a specific remodulation of EC as a function of age during early childhood (2–6 years old), also show significantly higher values in 7 to 18 years old individuals with ADHD. Together with these findings, it can thus be speculated that altered age-related trajectories in EC may represent the presence of a delayed or missed neurodevelopmental milestone in these individuals (Dark et al., 2018; Hannigan et al., 2021).

The Left and Right Executive Control networks exhibited significant but opposite correlations between EC and age, shifting from a marked left dominance in centrality towards an interhemispheric balance. Interestingly, this remodeling was not reflected by interhemispheric coordination, demonstrating how neurodevelopment drives different trajectories between inter- and intra-network connectivity patterns. The divergent development of the Left and Right Executive Control networks seems to be supported by evidence of white matter asymmetry, differences in functional interhemispheric connectivity, and reports of lateralized neural correlates for executive tasks (Asanowicz et al., 2012; Vallesi, 2012, 2021; Yin et al., 2013).

The dorsal and ventral DMN also showed significant but opposite correlations between EC values and age (positive for the ventral DMN, negative for the dorsal DMN). These findings might be interpreted in light of recent research on the separate role of these two components of the DMN (Chen et al., 2017; Lee et al., 2021; Sethi et al., 2018), and of the overlap between these regions and previously described ventral and dorsal streams of language processing (Hickok & Poeppel, 2007; Klein et al., 2015; Middlebrooks et al., 2017; Saur et al., 2008; Tomasi & Volkow, 2020; Wylie & Regner, 2014). As recent research highlighted the role of non-linear, non-monotonic trajectories in the neurodevelopment of the functional connectome in the human brain (Gracia-Tabuenca et al., 2021), especially for attention-related networks (Damiani et al., 2020; Gracia-Tabuenca et al., 2021), the authors warrant further research on the topic.

For what concerns ADHD severity, EC was correlated with the ADHD Global Index Severity score, and with the Inattentive/Hyper-impulsive subdomain scores. These correlations were evaluated at the network level, and were statistically significant primarily in the High Visual network and the Cerebellum. The correlation between symptomatic scores and EC values in the High Visual network can be better interpreted when considering previous neuroimaging studies, which highlighted consistent alterations in cortical thickness and functional activity in the medial Occipital cortex of patients with ADHD (Castellanos & Proal, 2012; Dickstein et al., 2006; Proal et al., 2011). Current voxel-wise results also showed important differences in EC values, which extended to most of the posterior brain.

Similarly, the correlation between symptomatic scores and Cerebellar EC values is in full agreement with the prefrontal-striatal-cerebellar model of ADHD (Curtin et al., 2018; Goetz et al., 2014; Krain & Castellanos, 2006; Lantieri et al., 2010). The prefrontal-striatal-cerebellar model posits a cerebellar involvement underpinning executive functioning, when integrated with the frontoparietal network (Cortese et al., 2012; Miquel et al., 2019; Mulder et al., 2008), and a cerebellar contribution to motor control, when integrated with somatosensory areas (Cortese et al., 2012; Picazio & Koch, 2015). The effect of EC at the network level was transdiagnostic, and neurotypicals showed a significant correlation between EC values and hyper-impulsivity in the Sensorimotor cortex and Executive Control networks. EC may therefore be posited as a marker of dimensional psychopathology rather than a diagnostic classification tool. As both clinical accounts and current results showed a protective role for age, to the present day it is not possible to exclude a potential compensatory plasticity during adolescence and young adulthood. Furthermore, EC correlated with age similarly in the two groups, with no significant difference between groups in the age effect for voxel-wise analyses. However, the correlations EC showed with age in dorsal DMN, right Executive Control, Sensorimotor network, and Cerebellum were opposite to the ones between EC and ADHD symptoms. Moreover, EC was not significantly correlated with handedness or IQ at the network level, which might be interpreted as a specificity of this measurement for the clinical correlates of ADHD psychopathology.

Although all included participants were right-handed, a dimensional approach to hand dominance allowed for novel interpretations about the role of hand dominance in the interhemispheric coordination and functional lateralization of the brain. In particular, interhemispheric coordination—as measured by VMHC—was significantly correlated with right-hand dominance in the Language Network in the neurotypical sample. By contrast, the groups of patients with ADHD showed a higher correlation between VMHC and handedness across several networks (Basal Ganglia, Language, ventral DMN, Visuospatial Networks). These findings show similar patterns to the high inter-participant and task-specific variability of lateralization in language processing areas (Cotosck et al., 2021; Gurunandan et al., 2020; Olulade et al., 2020; Vigneau et al., 2011), where marked functional lateralization is not clearly correlated to better performance. In turn, ventral DMN is central not only for sustained-attention (Sormaz et al., 2018) or goal-oriented behavior (Murphy et al., 2018; Spreng, 2012), but also for semantic fluency, entailing both cognition and memory (Martin et al., 2021). Consequently, in comparison to EC, VMHC rather seemed involved as a transdiagnostic marker of functioning in cognitive, verbal, or semantic tasks. In fact, VMHC correlated with IQ scores, but in a diverging manner between neurotypicals and ADHD. While neurotypicals showed negative correlations between VMHC and IQ in the Executive Networks, the group of participants with ADHD showed negative correlations in the Visuospatial Network only. Of special interest, previous studies described an interaction between auditory and visual processing, with reports suggesting the existence of a dual interplay between these processes, and the emergence of both interaction and segregation in brain areas related to these functions during late neurodevelopment (Berto et al., 2021). Moreover, studies have shown altered sensory processing in ADHD for what visual and auditory processing are concerned (Dunn & Bennett, 2002; Ghanizadeh, 2011; Schulze et al., 2021). Current results could then partially explain these findings in light of a divergent neurodevelopment between neurotypicals and individuals with ADHD. In fact, although VMHC was consistently and negatively correlated with age in both healthy controls and participants with ADHD, behavioral and cognitive functioning seemed to correlate with different brain networks in the two groups.

### Limitations

Although the included sample size was significantly high, further studies are needed in order to increase generalizability of results. Included participants ranged between 7 and 18 years old, thus warranting caution in interpreting results in light of an adult population. Although a dimensional approach to handedness allowed for a novel interpretation of results, further studies including both left and right-handed individuals are needed before definitive conclusions about the potential role of VMHC in determining interhemispheric coordination as a function of performance. Due to the explorative nature of the network-level analysis, several trends with uncorrected p between 0.05 and 0.01 were also reported, avoiding to perform more stringent corrections in order to reduce the risk of false negatives. The role of motion was controlled for with extensive preprocessing measures and controlled for both in quality evaluations of individual scans and group differences at the group level, however the authors warrant caution in drawing conclusions from a single study.

## Conclusions

EC was significantly higher in ADHD in respect to neurotypicals in the left inferior Frontal lobe, Lingual gyri, Peri-Calcarine cortex, superior and middle Occipital lobes, right inferior Occipital lobe, right middle Temporal gyrus, Fusiform gyri, bilateral Cuneus, right Precuneus, and Cerebellum. The current study suggested the specificity of EC as a correlate of ADHD psychopathology as assessed through the Conners’ Parent Rating Scale. VMHC was not found to be significantly different between participants with ADHD and neurotypicals, but a specific correlation was found between VMHC and handedness or IQ at the network level, suggesting a role of interhemispheric coordination in verbal or semantic associated areas and overall performance. Although all VMHC measures were negatively correlated with age in both healthy controls and participants with ADHD, behavioral and cognitive functioning correlated with different brain networks in the two groups. The authors interpreted this finding as further evidence of neurodivergence in ADHD. Finally, the authors discussed the complex relationship between EC, ADHD symptoms and age. Age significantly correlated (either positively or negatively) with the centrality of several brain networks. Brain networks where EC significantly correlated with clinical severity scores also exhibited opposite correlation coefficients between EC and age.

## Supplementary Information

Below is the link to the electronic supplementary material.

Supplementary file1 (DOCX 155 kb)

## Data Availability

The datasets generated during the current study are available from the corresponding author on reasonable request.

## Abbreviations

ADHD: Attention deficit / hyperactivity disorder
AUC: Area under the curve
BOLD: Blood oxygen level dependent signal
DMN: Default mode network
EC: Eigenvector centrality
LECN: Left executive control network
FC: Functional connectivity
fMRI: Functional magnetic resonance imaging
MNI: Montreal neurological institute
MRI: Magnetic resonance imaging
RECN: Right executive control network
TYP: Neurotypicals
VMHC: Voxel-mirrored homotopic connectivity

## Declarations

This study used a shared neuroimaging dataset from the UCLA Consortium for Neuropsychiatric Phenomics, all analyses conformed with the ethical standards as laid down in the 1964 Declaration of Helsinki and its later amendments. All participants consented to participate in the study and for future results to be published.
