# Supplementary material for: Centrality and interhemispheric coordination are related to different clinical/behavioral factors in attention deficit/hyperactivity disorder: a resting-state fMRI study
Source: Brain Imaging Behav. 2022 Jul 21;16(6):2526–42. doi: 10.1007/s11682-022-00708-8 (PMC9712307; doi:10.1007/s11682-022-00708-8)
Supplement: Supplementary file 1 — Supplementary file1 (DOCX 155 kb) [file 11682_2022_708_MOESM1_ESM.docx]

**Supplementary Materials**

**Table S1 – MNI coordinates of results. Synthetic account of surviving voxels at FDR-corrected-p 0.05**

| **% of voxels accounted for** | **Overlapping Anatomical Structure** | **Atlas code** |
| --- | --- | --- |
| 4.8% | Left Peri-Calcarine Cortex | 43 |
| 4.4% | Right Lingual Gyrus | 48 |
| 4.3% | Right Cerebellum (VIII) | 104 |
| 4.0% | Right Cerebellum (Crus 1) | 92 |
| 3.9% | Right Peri-Calcarine Cortex | 44 |
| 3.8% | Left Lingual Gyrus | 47 |
| 3.2% | Right Cerebellum (Crus 2) | 94 |
| 3.2% | Right Middle Occipital Gyrus | 52 |
| 2.6% | Right Cerebellum (VI) | 100 |
| 2.5% | Left Cerebellum (VIII) | 103 |
| 2.5% | Right Middle Temporal Gyrus | 86 |
| 2.4% | Left Cerebellum (VI) | 99 |
| 2.3% | Left Cuneus | 45 |
| 2.2% | Left Cerebellum (Crus 2) | 93 |
| 2.1% | Left Cerebellum (Crus 1) | 91 |
| 1.9% | Right Inferior Occipital Gyrus | 54 |
| 1.8% | Right Cuneus | 46 |
| 1.7% | Right Fusiform Gyrus | 56 |
| 1.6% | Right Precuneus | 68 |
| 1.4% | Left Superior Occipital Gyrus | 49 |
| 1.3% | Left Middle Occipital Gyrus | 51 |
| 1.2% | Left Precuneus | 67 |
| 1.2% | Right Superior Occipital Gyrus | 50 |
| 1.0% | Left Fusiform Gyrus | 55 |

*Note: intersection with atlas CA_ML_18_MNI; 75.8% of voxels accounted for. Regions < 1% not reported*

**Figure S2 - Violin Plot, distribution differences per group, mean FD value**

**
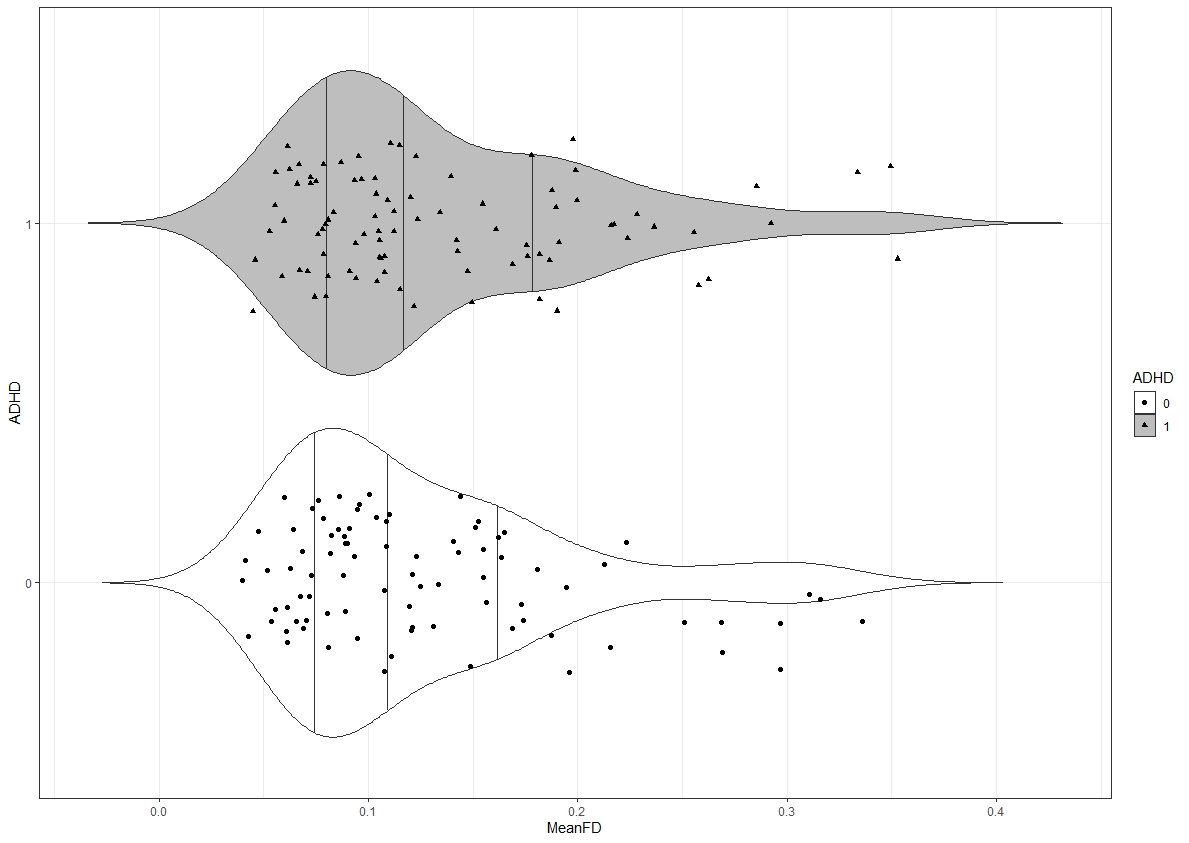
**

*Note: Violin plot divided per quartile of distribution (25, 50, 75 percentiles).*

*0 neurotypicals
1 ADHD participants*
